# Novel Primer Design for Significantly Reducing Fluorescent Interferences in the Synthesis of DNA-Templated Copper Nanoclusters for the Detection of the HLA-B*5801 Gene

**DOI:** 10.1021/acssensors.4c03116

**Published:** 2025-03-25

**Authors:** Ke-Peng Lai, Bo-Yu Liu, Wei-Lung Tseng, Hwang-Shang Kou, Chun-Chi Wang

**Affiliations:** † School of Pharmacy, College of Pharmacy, Kaohsiung Medical University, Kaohsiung, Taiwan 807, ROC; ‡ Department of Chemistry, National Sun Yat-sen University, Kaohsiung, Taiwan 804, ROC; § Department of Medical Research, Kaohsiung Medical University Hospital, Kaohsiung, Taiwan 807, ROC; ∥ Drug Development and Value Creation Research Center, Kaohsiung Medical University Hospital, Kaohsiung, Taiwan 807, ROC

**Keywords:** copper nanoclusters, high GC content PCR, HLA-B*5801 gene, fluorescence, visualization analysis

## Abstract

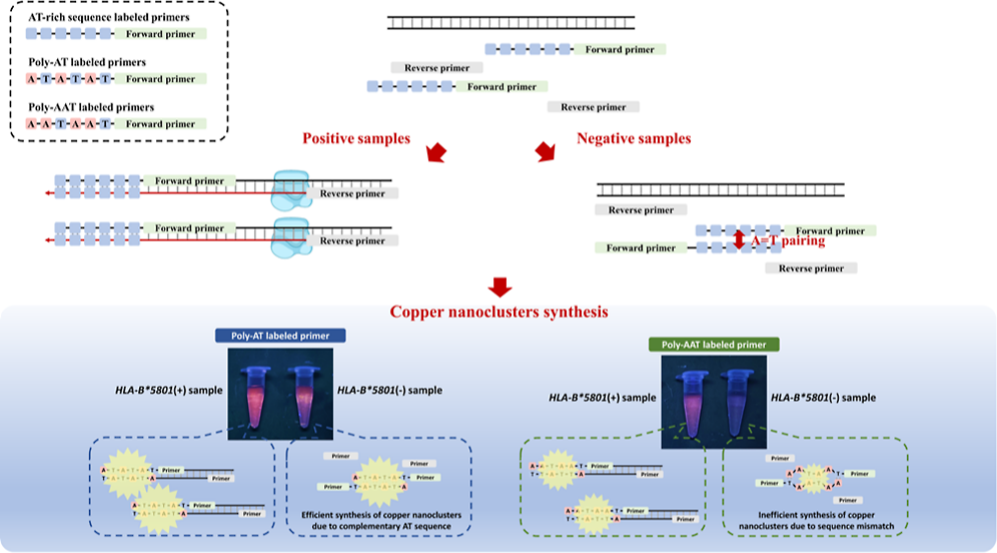

The optimal sequence for synthesizing copper nanoclusters is a promising research area. Initially, random dsDNA sequences yielded low fluorescence intensity, which constrained visual detection under UV light. Poly-AT dsDNA sequences later produced visible fluorescence, but it caused significant interference in negative samples when combined with gene amplification techniques. This interference occurs because the single-stranded poly-AT primer can self-anneal into a double-stranded AT sequence, efficiently synthesizing copper nanoclusters. To mitigate this, we designed a poly-AAT sequence at the primer’s 5′ end, creating a single base pair mismatch every three nucleotides during self-annealing. This adjustment reduced synthesis efficiency of copper nanoclusters in negative samples, improving the visual distinction between negative and positive results. We applied this method to identify the HLA-B*5801 gene, thereby demonstrating its efficacy even within a GC-rich region of human genomic DNA. Our method showed 100% agreement with a commercial qPCR kit, with results distinguishable under UV light. We conclude that the poly-AAT sequence is more suitable for integrating copper nanoclusters synthesis with nucleic acid amplification detection techniques, with potential applications in microelectronics, biosensing, and catalysis.

In recent years, metal nanomaterials had attracted considerable scholarly attention due to their broad applicability in microelectronics, biosensing, catalysis, and biomedicine.^1−4^ These materials can be synthesized using biomolecules, including DNA, proteins, amino acids, and thiol derivatives.^5−8^ Among these biomolecules, DNA is particularly advantageous as a template for the synthesis of metal nanoclusters, owing to its nanoscale architecture and electrostatic characteristics. For several decades, DNA-templated metal nanoclusters had been utilized in DNA analysis and DNA probe-based biosensors.^9−12^ In terms of cost-effectiveness and synthesis efficiency, copper nanoclusters are among the most promising candidates, capable of being synthesized within 5 min at room temperature.^13^ Initially, random double-stranded DNA (dsDNA) sequences were employed for the synthesis of copper nanoclusters; however, the resulting fluorescence intensity was low and difficult to detect under ultraviolet (UV) light.^8^ Currently, more efficient DNA sequences for the synthesis of copper nanoclusters had been identified: poly-AT for double-stranded DNA and poly-T for single-stranded DNA.^14−16^ The fluorescence intensity of copper nanoclusters synthesized using AT-rich sequences is sufficiently high to be observed under UV light, leading to their extensive use in recent research endeavors.

By incorporating the desired sequence at the 5′ terminus of the primers, the resulting polymerase chain reaction (PCR) amplicons can integrate an additional artificial sequence, thereby facilitating the synthesis of copper nanoclusters.^17^ However, the incorporation of a poly-AT sequence at the 5′ end of PCR primers results in only a marginal increase in the fluorescence signal of positive samples, with an enhancement of no more than one-fold when compared to negative samples.^18^ Furthermore, concerns had been raised regarding the potential interference of the artificial sequence attached to the 5′ end of the primers with the PCR process during the insertion into double-stranded DNA. These limitations had impeded the application of copper nanoclusters in PCR-based biosensors.

Severe cutaneous adverse reactions (SCARs) are a consequence of an immune overreaction induced by certain medications.^19,20^ The mortality rate associated with SCARs is approximately 30%.^21−23^ A significant correlation had been established between SCARs and human leukocyte antigen (HLA) genes. For instance, allopurinol is a first-line urate-lowering therapy for hyperuricemia but can induce severe cutaneous adverse reactions (SCARs).^24^ The HLA-B*5801 gene, found on chromosome 6, is strongly linked to allopurinol-induced SCARs, making its detection essential for prevention.^25,26^ Due to the considerable diversity and similarity present among HLA genes, these genes are predominantly analyzed utilizing techniques such as melting curve analysis, real-time PCR, sequence-specific oligonucleotide probing (SSO), and sequencing-based typing (SBT).^27,28^ These methodologies are employed to mitigate the potential for false detections. However, these methods necessitate expensive equipment and skilled personnel, rendering them impractical for laboratories with limited resources.

To address the aforementioned limitations, we developed a specialized sequence at the 5′ end of the specific primers. The resulting PCR products were subsequently utilized for the synthesis of copper nanoclusters. By employing the designed sequence, the fluorescent signal from negative samples can be significantly diminished. In contrast, positive samples yield amplicons that contain the specialized sequence, thereby facilitating the efficient synthesis of copper nanoclusters. The resultant fluorescent differences between positive and negative results are markedly enhanced and can be distinguished by the naked eye. For our demonstration, we selected HLA-B*5801 as the target, which is located within a region of human genomic DNA characterized by high GC content (>70%). This selection illustrates the applicability of our strategy across a diverse range of PCR scenarios.

## Experimental Section

### Chemicals and Reagents

PrimeSTAR GXL PCR kit, which includes 5 × PrimeSTAR GXL Buffer (Mg^2+^ plus), 2.5 mM dNTPs, and 1.25 U/μL PrimeSTAR GXL DNA Polymerase, was procured from Takara Biotechnology (Japan). The primers and templates designed for this study, as detailed in Table S1, were synthesized by Genomics BioSci & Tech (New Taipei City, Taiwan). A 100 bp DNA ladder was supplied by Antec Bioscience Inc. (Taipei, Taiwan). The 5 × Tris-borate-EDTA (TBE) buffer was obtained from Protech Technology Enterprise (Taipei, Taiwan). YO-PRO-1 iodide was purchased from Molecular Probes (Invitrogen Detection Technologies, Eugene, OR, USA). Copper sulfate, sodium l-ascorbate, 3-morpholinopropane-1-sulfonic acid (MOPS), and poly(ethylene oxide) (PEO) with a molecular weight of approximately 8,000,000 were supplied by Sigma-Aldrich (St. Louis, MO, USA). Sodium chloride (NaCl) and sodium hydroxide (NaOH) were acquired from E. Merck (Darmstadt, Germany). Deionized water utilized in this study was produced using a Milli-Q water system (Millipore, Milford, MA, USA).

### Preparation of PCR Reagents and Reacting Condition

In this study, all PCRs were performed utilizing a thermocycler (Biometra T Professional, Germany). Touchdown PCR was executed in accordance with the protocol detailed in Table S2. The final composition of the 25 μL PCR reaction mixture comprised 1× PCR buffer, 200 μM dATP, 200 μM dTTP, 200 μM dCTP, 200 μM dGTP, 0.2 μM of each forward and reverse primer, and 50 ng of genomic DNA (gDNA) sample. The concentrations of each reagent were referenced from the manufacturer’s instructions.

The results of the PCR were subsequently validated through capillary gel electrophoresis (CGE) using equipment from Beckman Instruments (Fullerton, CA, USA). A gel buffer composed of 1% poly(ethylene oxide) (PEO) and 2× TBE buffer was employed to separate amplicons of varying lengths. For DNA staining, 1 μL of YO-PRO-1 was mixed with 1 mL of the gel buffer. A laser-induced fluorescence (LIF) detector, utilizing an excitation wavelength of 488 nm and an emission wavelength of 520 nm, was employed to monitor the separation results during the assay. A DNA ladder (100 bp) served as a reference for comparing and estimating the lengths of the amplicons. Prior to CGE analysis, the PCR products were diluted 1000-fold. Samples were injected by applying −8 kV to the mixture for a duration of 5 s and were subsequently separated at −10 kV for 15 min. The temperature was maintained at 25 °C throughout the procedure.

### Different Primer Sequences for DNA-Templated Ccopper Nnanoclusters Synthesis

We designed various sequences extending from HLA-B*5801-specific primers (see Table S1). Each primer was modified to include a 45-mer extension composed of poly-AT, poly-AAT, poly-ATT, poly-T, or a random sequence. Following the PCR of HLA-B*5801 samples, dsDNA amplicons were generated, which facilitated the synthesis of dsDNA-templated copper nanoclusters. Additionally, we compared the fluorescence signals associated with different lengths of AAT on the specific primers. The results were analyzed using a Hitachi F-4500 fluorescence spectrometer (Hitachi, Japan), with excitation and emission wavelengths set at 350 and 570 nm, respectively. Both the excitation and emission slits were configured to 5 nm, the scanning speed was maintained at 1200 nm/min, and a photomultiplier tube (PMT) voltage of 700 V was employed throughout the experiment. High-resolution transmission electron microscopy (HRTEM) images were obtained using a Talos F200X G2 field emission scanning transmission electron microscope (Thermo Fisher Scientific, USA) to confirm the formation of the copper nanoclusters. Dynamic light scattering (DLS) analysis were conducted using an ELSZ-2000 (Otsuka, Japan). Scanning electron microscopy (SEM) was performed with a JSM-6700F (JEOL, Japan). Furthermore, powder X-ray diffraction (XRD) analysis was executed using a D2 Phaser diffractometer (Bruker, Germany).

### Synthesis of Ccopper Nnanoclusters

This study investigates the optimal conditions for the synthesis of poly-AAT-templated copper nanoclusters. The final synthesis conditions were established using a 200 μL reaction mixture that included 25 μL of PCR product, 300 μM copper sulfate, and 1500 μM sodium ascorbate, all within a 10 mM MOPS buffer at pH 7.5. The reaction mixture was incubated at room temperature for 3 min before fluorescence detection.

### Sensitivity, Specificity Assay in Real Sample and Visualized Detection

Genomic DNA samples were obtained from participants at Kaohsiung Medical University Chung Ho Hospital, located in Kaohsiung, Taiwan. Blood specimens were processed utilizing the ZEJU genomic DNA extraction kit (ZEJU Co., Ltd., Taiwan). The resulting DNA samples demonstrated an optical density (OD) ratio of 260/280 ranging from 1.8 to 2.0, as determined by a U-2900 spectrophotometer (Hitachi, Japan). These samples were subsequently diluted to a concentration of 50 ng/μL. The genotypes of all samples were verified using a qPCR kit (PharmiGENE, Taiwan) on an Applied Biosystems 7500 system (ThermoFisher, USA). A standard curve was generated to establish the detection limit of the employed methodology.

## Results and Discussion

### Analysis Results of Different Primer Sequences

The primary aim of this research was to determine the most suitable primer sequence for the synthesis of DNA-templated CuNCs after PCR. Previous studies had indicated that poly-AT serves as a more efficient dsDNA template;^15,29^ however, its application in PCR is hindered by significant interference from blank samples. It is posited that this interference occurs due to the ability of single-stranded poly-AT to function as its own complementary sequence. This property allows the primers to self-anneal, resulting in the formation of a double-stranded poly-AT template. This template is capable of synthesizing copper nanoclusters efficiently, even in the absence of successful amplification. As a result, the fluorescence signal of the PCR amplicons is only marginally elevated compared to that of the blank samples.^18^

**Figure 1:**
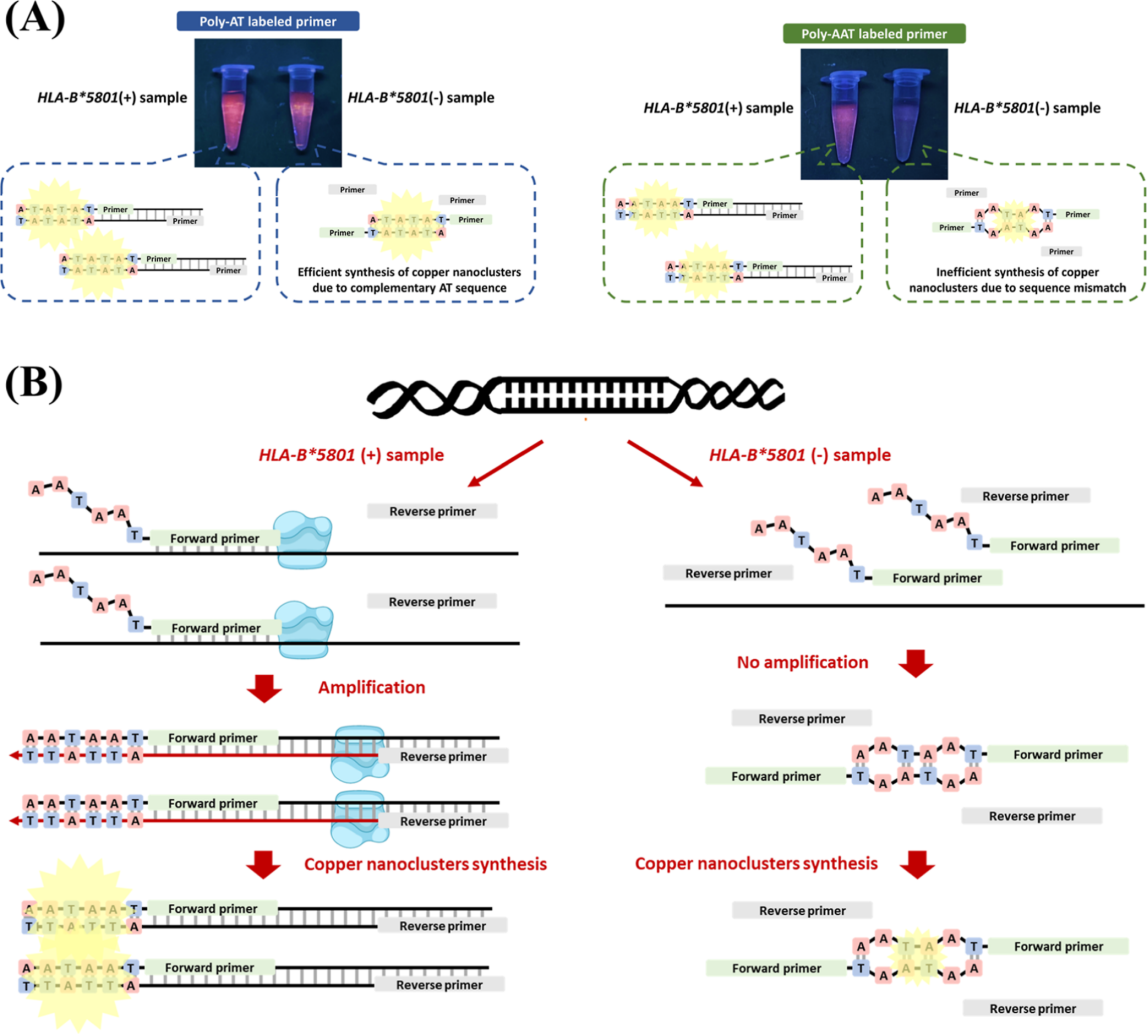
Our method for detecting the HLA-B*5801 gene minimizes interference during copper nanocluster synthesis. (A) This section presents the theoretical framework and visual results showing reduced fluorescence interference with the poly-AAT sequence compared to the poly-AT sequence. (B) This section describes how amplicons are generated using the poly-AAT sequence.

The primary distinction between gene amplification and primer self-annealing lies in the fact that the sequences generated through gene amplification exhibit complete complementarity. Building upon this understanding, we formulated the concept of integrating the properties of AT-rich sequences, which demonstrate enhanced efficiency for copper nanoclusters, with the observation that the synthesis efficiency of DNA-templated copper nanoclusters is adversely affected by nucleotide mismatches.^8^ As a result, we inserted an adenosine between the poly-AT sequence and formed poly-AAT sequence that guarantees the occurrence of a mismatch in every three nucleotides during the self-annealing of primers. This modification resulted in a reduced synthesis efficiency of copper nanoclusters, consequently leading to diminished fluorescence in negative samples (Figure 1A). As for positive samples, the polymerase generates fully complementary double-stranded poly-AAT sequences, facilitating the synthesis of copper nanoclusters (Figure 1B).

**Figure 2:**
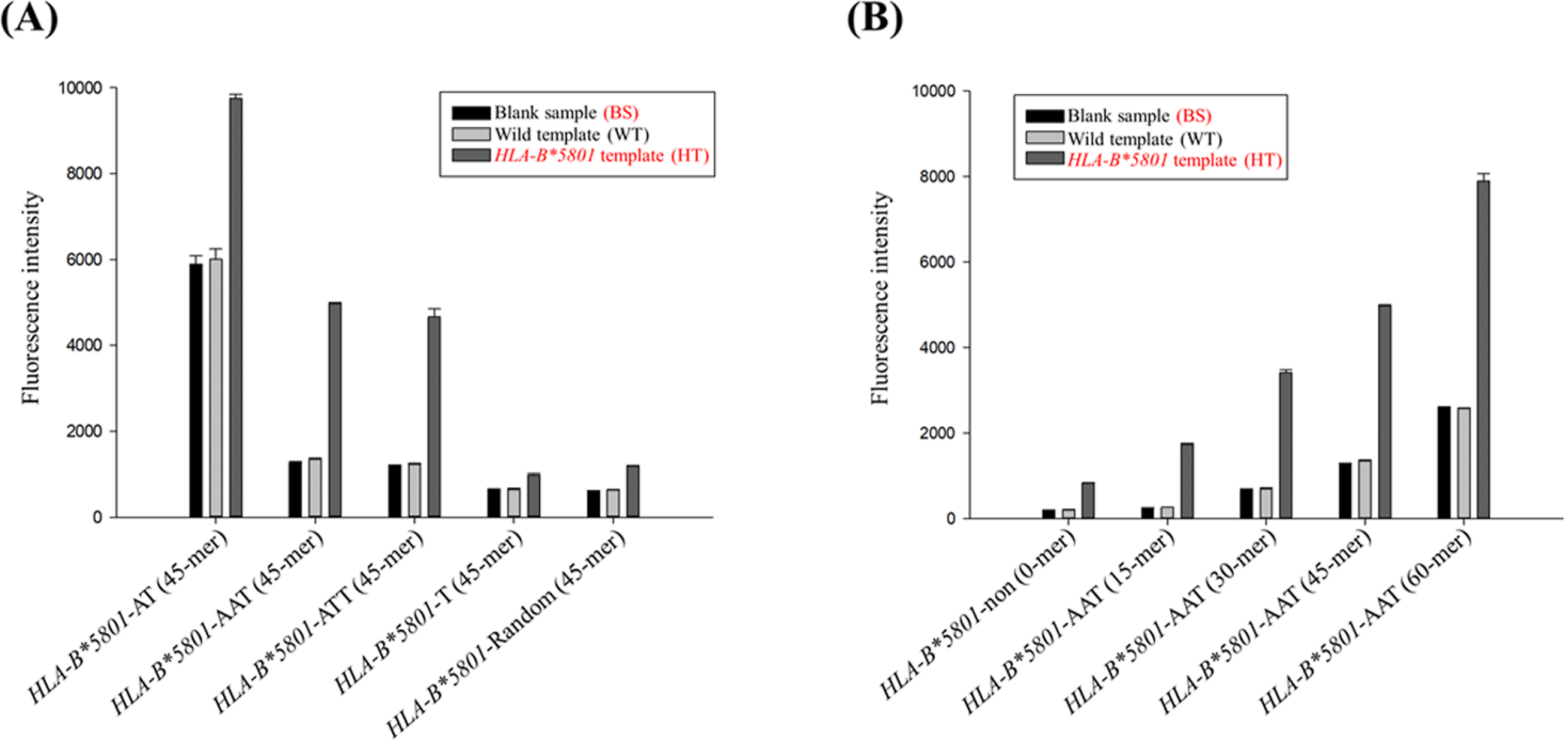
Evaluating the optimal sequence for our assay. (A) Fluorescence differences among blank sample (BS), wild template (WT), and HLA-B*5801 template (HT) with various additional primer sequences (*n* = 3). (B) Fluorescence differences among blank sample (BS), wild template (WT), and HLA-B*5801 template (HT) with different lengths of poly-AAT sequences (*n* = 3). [copper nanoclusters were synthesized using MOPS buffer (10 mM, 150 mM NaCl, pH 7.5), 300 μM copper sulfate, and 1500 μM sodium ascorbate].

Consequently, we incorporated the novel poly-AAT sequence at the 5′ end of our HLA-B*5801 specific primer and compared the detection effectiveness with commonly used poly-AT,^18^ poly-T^30^, and random sequences^17^ in gene amplification-related literature (Figure 2A). As expected, the poly-AT sequence showed the highest fluorescence with the HLA-B*5801 template, but blank sample and wild template also exhibited elevated fluorescence, which hindered accurate genotype identification. In contrast, the poly-AAT sequence maintained acceptable fluorescence with the HLA-B*5801 template while significantly reducing fluorescence in blank sample and wild template, indicating its superiority over the poly-AT sequence for PCR assays. Similar outcomes were observed with the poly-ATT sequence, which operates under the same mechanism by creating a mismatch in every three nucleotides during self-annealing in blank sample and wild template (Figure S1). Although the poly-T sequence demonstrates superior efficiency in the synthesis of copper nanoclusters from ssDNA, its fluorescence intensity is significantly lower compared to that of double-stranded sequences composed of both adenine and thymine.^29^ Similar to the poly-T sequence, the copper nanoclusters synthesized from random sequence do not have sufficient fluorescence intensity to be observed by the naked eye under UV light.

To further ascertain that the significant reduction in fluorescence of negative samples is attributable to mismatches caused by using poly-AAT labeled primers while self-annealing, rather than simply due to the sequences themselves, we conducted additional experiments to elucidate the underlying mechanism. We conducted a comparative analysis of the PCR results utilizing 0.2 μM poly-AAT, 0.2 μM poly-ATT, and 0.1 μM of each poly-AAT and poly-ATT labeled primers (Figure S2A). As previously indicated, the individual poly-AAT and poly-ATT labeled primers are capable of generating mismatches during the self-annealing process (Figure S2B,C). Conversely, when these two primers are used in combination, they demonstrate complete complementary annealing (Figure S2D). The fluorescent intensity of both the blank and wild templates exhibits a significant increase when the combined primers are employed. Therefore, it is confirmed that the notable reduction in fluorescence observed in negative samples associated with poly-AAT labeled primers is attributable to the mismatches generated during the self-annealing of the primers.

Subsequently, we determined the optimal length for the extended poly-AAT sequence by conducting a comparative analysis of sequences containing 0, 15, 30, 45, and 60 AAT repeats (Figure 2B). The fluorescence intensity of the blank, wild, and HLA-B*5801 templates exhibited an increase corresponding to the length of the poly-AAT sequence. Notably, the fluorescence intensity of sequences containing fewer than 15 AAT repeats was insufficient for reliable visual detection (Figure S3). Additionally, considering the expense associated with labeling longer sequences, we ultimately selected 15 AAT repeats as the primer sequence for the entirety of this study.

### Optimization for copper nanoclusters Synthesis

**Figure 3:**
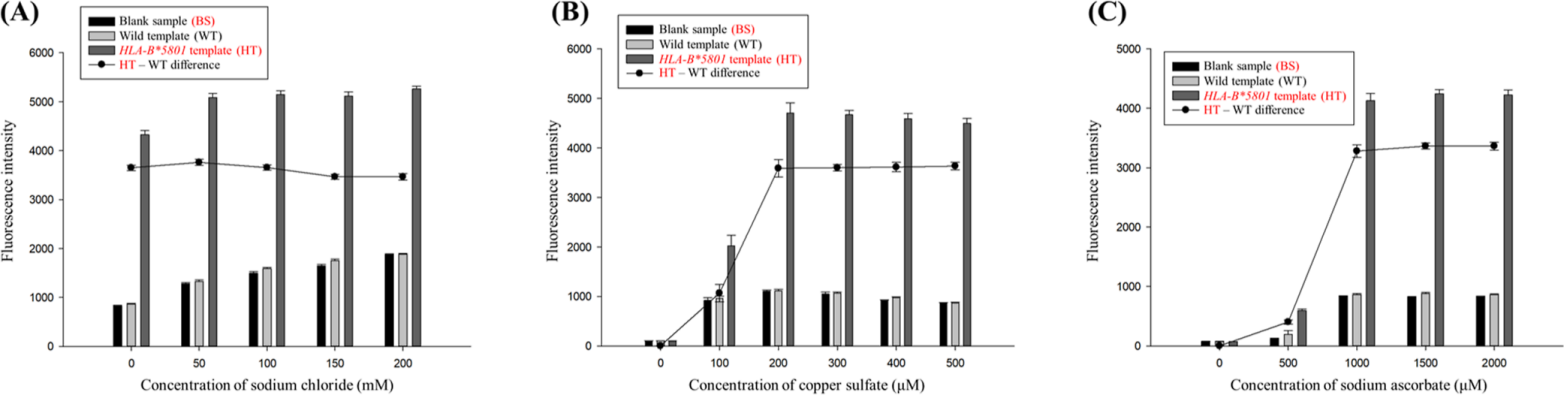
Optimizing synthesis conditions for poly-AAT-templated copper nanoclusters and their properties. (A) Effect of sodium chloride concentration on PCR products of HLA-B*5801-45AAT primers (*n* = 3). (B) Impact of copper sulfate concentration on PCR products of HLA-B*5801-45AAT primers (*n* = 3). (C) Influence of sodium ascorbate concentration on PCR products of HLA-B*5801-45AAT primers (*n* = 3).

To date, there have been no studies focused on optimizing the synthesis conditions for the poly-AAT sequence. Consequently, we devised a series of experiments aimed at identifying the optimal conditions for the synthesis of poly-AAT-templated copper nanoclusters. Initially, we discussed the conditions of the MOPS buffer solution. The MOPS buffer provides a conducive environment for the synthesis of copper nanoclusters and has been extensively utilized in several related studies.^8,18,29,30^ The most commonly employed buffer in previous research consists of 10 mM MOPS and 150 mM NaCl. However, sodium chloride can affect DNA hybridization, consequently impacting the efficiency of copper nanocluster synthesis in negative samples. Therefore, we examined the effects of sodium chloride at concentrations of 0, 50, 100, 150, and 200 mM. As illustrated in Figure 3A, the fluorescence intensity of the negative samples increased with the concentration of sodium chloride. Although a concentration of 50 mM sodium chloride exhibited the greatest fluorescence difference between HLA-B*5801 negative and positive samples, we chose to exclude sodium chloride from our experiments. This decision was made to minimize the fluorescence interference arising from HLA-B*5801 negative samples. Furthermore, it is possible that the PCR reagents already contained an adequate amount of salt necessary for the synthesis of copper nanoclusters.

Copper sulfate serves as a widely utilized precursor in the synthesis of copper nanoclusters.^9−12^ In the present study, we examined the effects of varying concentrations of copper sulfate—specifically 0, 100, 200, 300, 400, and 500 μM—on fluorescence intensity to determine the optimal conditions for the synthesis of copper nanoclusters. As illustrated in Figure 3B, fluorescence intensity reached its maximum at a concentration of 200 μM; however, the corresponding standard deviation was significantly higher. Consequently, a concentration of 300 μM copper sulfate was consistently employed throughout the experimental procedures.

Sodium ascorbate serves as a reducing agent in the synthesis of copper nanoclusters, facilitating the reduction of Cu(II) to Cu(0) on DNA templates.^8^ It is thus imperative to identify the optimal concentration of sodium ascorbate for the effective synthesis of copper nanoclusters. In our experiments, we tested sodium ascorbate concentrations of 0, 500, 1000, 1500, and 2000 μM. The fluorescence intensity measurements indicated that synthesis efficiency appeared to plateau when concentrations exceeded 1000 μM (Figure 3C). In light of the photodegradation properties of sodium ascorbate, we ultimately determined that a concentration of 1500 μM sodium ascorbate would provide optimal stability for the subsequent experiments.

### Properties of Poly-AAT-Templated copper nanoclusters

**Figure 4:**
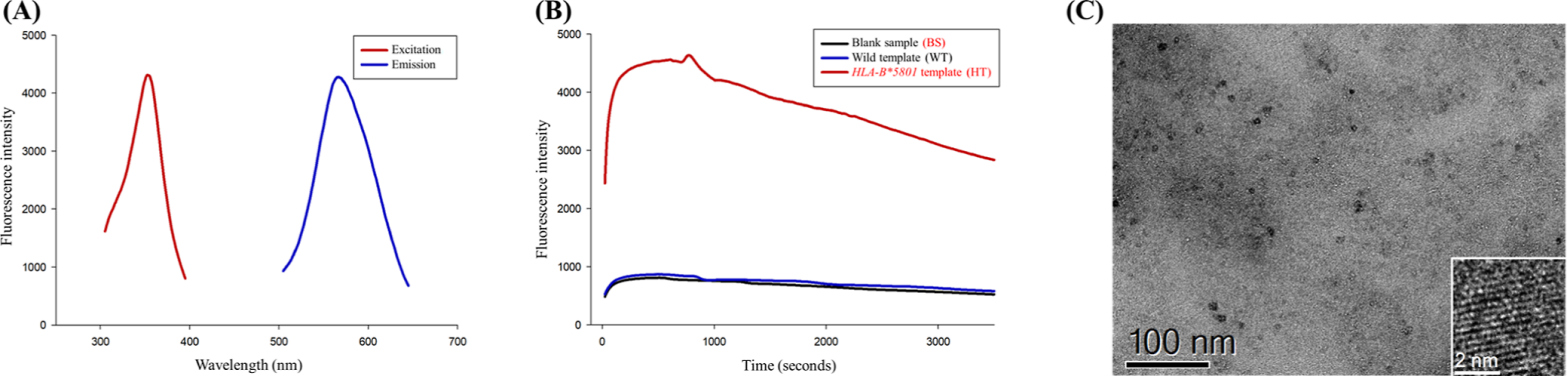
Fundamental properties of poly-AAT-templated copper nanoclusters synthesized under optimized conditions. (A) Excitation (red) and emission (blue) wavelength scans of poly-AAT-templated copper nanoclusters. (B) Fluorescence time scans of blank sample (BS), wild template (WT), and HLA-B*5801 template (HT). (C) HRTEM image of copper nanoclusters synthesized with poly-AAT sequence.

We conducted a comprehensive analysis of the fundamental properties of copper nanoclusters synthesized under optimized conditions. The most pronounced fluorescence was observed upon excitation at approximately 350 nm, with peak emission occurring at around 570 nm (Figure 4A). The copper nanoclusters demonstrated considerable stability across the blank sample, wild template, and HLA-B*5801 template for 10 min, after which a gradual decline in fluorescence intensity was observed (Figure 4B). This observation suggests that the stability of the synthesized copper nanoclusters is adequate for analytical purposes.

The HRTEM image presented in Figure 4C demonstrates that the poly-AAT-templated copper nanoclusters possess a spherical morphology, with diameters ranging from approximately 2–4 nm. Dynamic light scattering (DLS) measurements support these observations, indicating that the hydrodynamic diameter of the copper nanoclusters is approximately 2–5 nm (Figure S4A). However, during scanning electron microscopy (SEM) imaging, some aggregation was noted, as illustrated in Figure S4B. Additionally, the powder X-ray diffraction (XRD) analysis spectrum revealed that the signals associated with copper were markedly weaker (Figure S4C). This observation aligns with previous studies involving other DNA-templated copper nanoclusters.^11^ The broadening of the diffraction peaks toward the baseline further indicates the small size of the copper clusters.^31^

### Usage of Touchdown PCR

**Figure 5:**
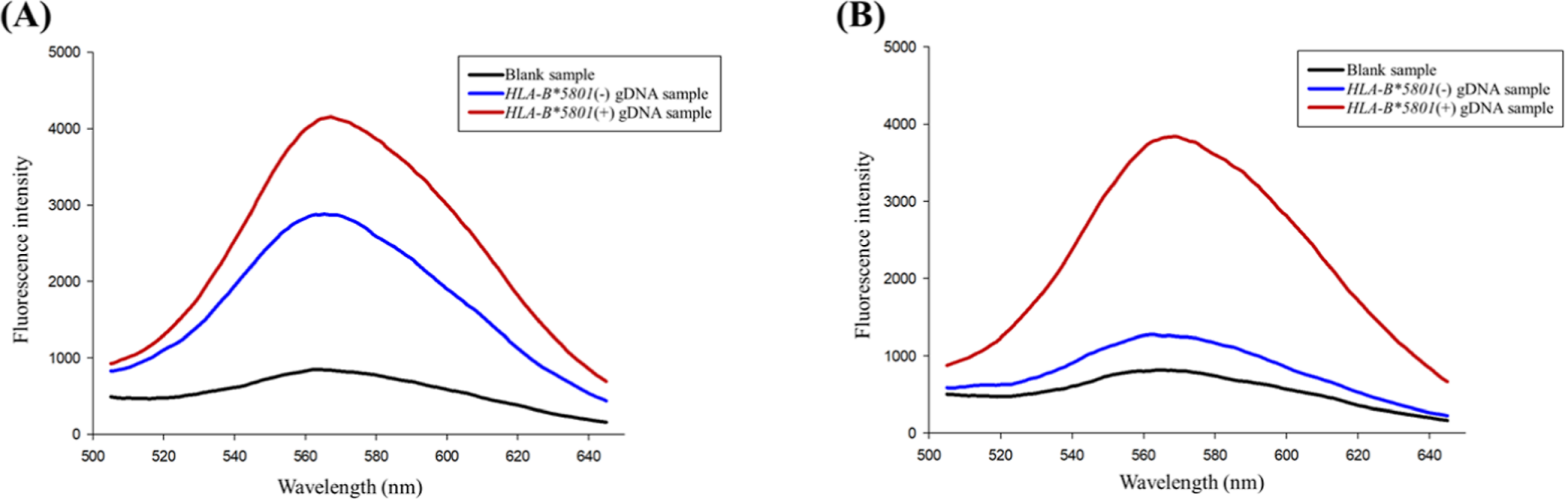
Application of touchdown PCR in gDNA samples. (A) The fluorescence diagram of copper nanoclusters synthesized with blank, HLA-B*5801(−), and HLA-B*5801(+) gDNA PCR products at a fixed annealing temperature of 63 °C. (B) The fluorescence diagram of copper nanoclusters synthesized with blank, HLA-B*5801(−), and HLA-B*5801(+) gDNA via a touchdown PCR protocol.

Previous research indicates that the specificity of PCR can be substantially enhanced, and the yield improved, through the implementation of touchdown PCR in comparison to conventional PCR methods.^32−34^ Given the high degree of similarity observed among the DNA sequences of HLA variants, we conducted a comparative analysis of the fluorescence results obtained from both touchdown PCR and conventional PCR techniques. Our findings revealed that the use of standard PCR conditions (using a constant annealing temperature of 63 °C) may result in false-positive outcomes in certain gDNA samples (Figure 5A). In contrast, the application of touchdown PCR significantly mitigated such interference (Figure 5B) and was subsequently integrated into our experimental design.

Subsequently, we employed capillary gel electrophoresis to verify whether the length of our PCR products conformed to expectations. The length of the PCR amplicons generated using the poly-AAT primer was determined to be 399 base pairs. As illustrated in Figure S5, the capillary electrophoresis (CE) diagram of the HLA-B*5801(+) gDNA sample exhibited a distinct single peak signal at approximately 12 min. When compared to the 100 bp DNA ladder, the amplicons were estimated to be around 400 bp, which aligns with the anticipated results. In contrast, the blank sample and HLA-B*5801(−) gDNA samples displayed no discernible peaks on the electrophoresis diagram. The results indicated that the primers we developed, in conjunction with touchdown PCR, demonstrate a high level of specificity for the target gene segment and do not amplify other genomic regions.

### Real Sample Assay

**Figure 6:**
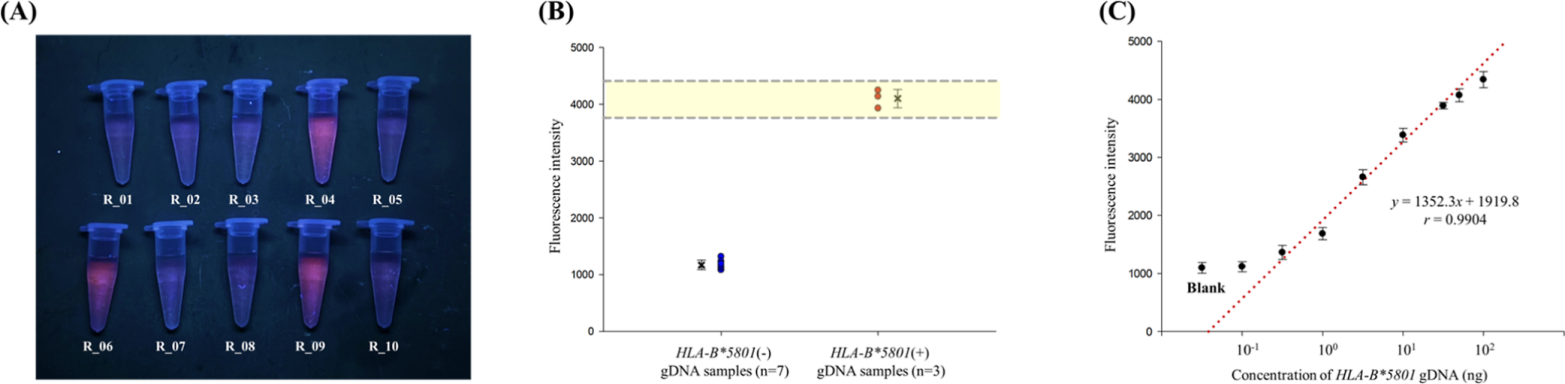
Application of our strategy to gDNA samples. (A) The fluorescence image of 10 gDNA samples under 365 nm UV light detected using our analysis method (*n* = 10). (B) The distribution dot plot of fluorescence intensity of HLA-B*5801(−), and HLA-B*5801(+) gDNA samples. (C) The calibration curve of HLA-B*5801 gDNA measured with optimized condition (*n* = 3).

In this study, we collected ten DNA samples from the peripheral blood of participants and confirmed their genotype using a qPCR kit (refer to Table 1). Among the ten samples analyzed, three exhibited a positive signal for HLA-B*5801 when exposed to UV light (Figure 6A). This finding indicates that the detection outcomes of our methodology are consistent with the results obtained from qPCR. We evaluated the fluorescence data and determined the positive fluorescence range for HLA-B*5801 by calculating the mean fluorescence intensity of the HLA-B*5801(+) samples and using two standard deviations as the margin of error (Figure 6B). Consequently, a fluorescence intensity range of 3781.89–4426.11 was established as indicative of HLA-B*5801 positivity.

**Table 1:** Fluorescence Intensity of Genotype of 10 gDNA Samples Confirmed with qPCR (*n* = 3)

sample ID	Ct_HLA-B*5801gene_	Ct_Housekeepinggene_	ΔCt^a^	qPCR results	our results	fluorescence intensity	SD value
R_01	32.301	24.597	7.704	–	–	1086.33	25.66
R_02	31.772	22.734	9.038	–	–	1275.67	38.89
R_03	31.792	21.530	10.262	–	–	1053.53	47.69
R_04	24.185	21.315	2.87	+	+	4234.67	87.36
R_05	34.707	21.490	13.217	–	–	1167.67	26.54
R_06	23.521	20.092	3.429	+	+	4216.67	129.95
R_07	34.897	20.924	13.973	–	–	1287.00	52.12
R_08	35.730	19.351	16.379	–	–	1193.00	55.05
R_09	23.424	19.608	3.816	+	+	4135.00	69.76
R_10	33.614	23.481	10.133	–	–	1141.33	28.31

a ΔCt = Ct_HLA-B*5801gene_ – Ct_Housekeepinggene_, ΔCt ≤ 7 is defined as HLA-B*5801 positive.

To assess the sensitivity of our methodology, a calibration curve was constructed utilizing standards of HLA-B*5801 gDNA, as depicted in Figure 6C. The linear regression equation was derived using concentrations ranging from 10^–5^ to 10^1.5^ ng. The observed linearity was deemed unsatisfactory, which may be attributed to the application of touchdown PCR, a technique that is not ideally suited for the quantitative assessment of target concentration.^31^ The limit of detection (LOD) was determined using the formula FL_Blank_ + 3 × SD_Blank_, where FL_Blank_ represents the average fluorescence intensity of the blank sample and SD_Blank_ denotes the standard deviation of the blank sample. The limit of detection (LOD) calculated for our method was determined to be 0.39 ng. However, considering factors such as sample consumption and the quality of visual results, a concentration of 50 ng of gDNA was deemed appropriate for the detection of real samples.

## Conclusions

In this study, we identified a more suitable sequence for the synthesis of copper nanoclusters compared to the poly-AT sequence utilized in PCR assays. By employing the poly-AAT sequence instead of the poly-AT sequence, we observed a significant enhancement in the fluorescence difference between HLA-B*5801(−) and HLA-B*5801(+) gDNA samples after PCR. The fluorescent results are visible when exposed to UV light. The limit of detection of our method was determined to be 0.39 ng, as measured using a fluorescence spectrometer. The efficacy of this method in HLA-B*5801 suggests its applicability even in high GC content gDNA PCR scenarios. The results of our study indicate multiple potential applications for this method. It can function as a fluorometric method applicable to a range of gene detection techniques and biosensors. Additionally, after synthesis, it may be employed as a nanomaterial in the fields of microelectronics, catalysis, and biomedicine.

## Supporting Information Available

The sequence of primers and template; touchdown PCR protocol the mechanism of poly-ATT; supplementary evidence of mismatching; fluorescence images obtained under UV light; additional characteristics of poly-AAT templated copper nanoclusters and capillary gel electrophoresis diagram (PDF)

## Abbreviations

SCARs: severe cutaneous adverse reactions
HLA: human leukocyte antigen
MOPS: 3-morpholinopropane-1-sulfonic acid
PEO: poly(ethylene oxide)
CGE: capillary gel electrophoresis
LIF: laser-induced fluorescence
HRTEM: high-resolution transmission electron microscope
DLS: dynamic light scattering
SEM: scanning electron microscopy
XRD: X-ray diffraction
