# Supplementary material for: Novel Primer Design for Significantly Reducing Fluorescent Interferences in the Synthesis of DNA-Templated Copper Nanoclusters for the Detection of the HLA-B*5801 Gene
Source: ACS Sens. 2025 Mar 25;10(4):2609–16. doi: 10.1021/acssensors.4c03116 (PMC12038829; doi:10.1021/acssensors.4c03116)
Supplement: Supplementary file 1 — se4c03116_si_001.pdf [file se4c03116_si_001.pdf]

Supporting Information

A Novel Primer Design for Significantly Reducing Fluorescent Interferences in the Synthesis of DNA-Templated Copper Nanoclusters for the Detection of the *HLA-B*5801* Gene

Ke-Peng Lai[a], Bo-Yu Liu[b], Wei-Lung Tseng[a,b], Hwang-Shang Kou[a], Chun-Chi Wang[a,c,d,]*

[a] School of Pharmacy, College of Pharmacy, Kaohsiung Medical University, Kaohsiung 807, Taiwan, ROC.

[b] Department of Chemistry, National Sun Yat-sen University, Kaohsiung 804, Taiwan, ROC.

[c] Department of Medical Research, Kaohsiung Medical University Hospital, Kaohsiung 807, Taiwan, ROC.

[d] Drug Development and Value Creation Research Center, Kaohsiung Medical University Hospital, Kaohsiung 807, Taiwan, ROC.

Corresponding authors:
Chun-Chi Wang, Ph.D.
School of Pharmacy, College of Pharmacy, Kaohsiung Medical University
100, Shi-Chuan 1st Rd., Kaohsiung 807, Taiwan
Fax: 886-7-3210683
Tel: 886-7-3121101 ext 2253
E-mail: chunchi0716@kmu.edu.tw

Table S1. The sequence of primers and templates utilized in this study.

Primer	Sequence (5’→3’)
***HLA-B*5801*-non (0-mer)**	GAACATGAAGGCCTCCGCG
***HLA-B*5801*-AAT (15-mer)**	AATAATAATAATAATGAACATGAAGGCCTCCGCG
***HLA-B*5801*-AAT (30-mer)**	AATAATAATAATAATAATAATAATAATAATGAACATGAAGGCCTCCGCG
***HLA-B*5801*-AAT (45-mer)**	AATAATAATAATAATAATAATAATAATAATAATAATAATAATAATGAACATGAAGGCCTCCGCG
***HLA-B*5801*-AAT (60-mer)**	AATAATAATAATAATAATAATAATAATAATAATAATAATAATAATAATAATAATAATAATGAACA<br>TGAAGGCCTCCGCG
***HLA-B*5801*-AT (45-mer)**	ATATATATATATATATATATATATATATATATATATATATATATAGAACATGAAGGCCTCCGCG
***HLA-B*5801*-ATT (45-mer)**	ATTATTATTATTATTATTATTATTATTATTATTATTATTATTATTATTGAACATGAAGGCCTCCGCG
***HLA-B*5801*-T (45-mer)**	TTTTTTTTTTTTTTTTTTTTTTTTTTTTTTTTTTTTTTTTTTTTTGAACATGAAGGCCTCCGCG
***HLA-B*5801*-Random (45-mer)**	GTCGTGATGAACGTATGAGCGTATGAGTATACTCATACGCTCATAGAACATGAAGGCCTCCGCG
**Reverse primer**	GCAGCCATACATCCTCTGGATGA
**Template**	**Sequence (5’→3’)**
**Wild**	CGACACCCAGTTCGTGAGGTTCGACAGCACGCCGCGAGTCCGAGAGGAGCCGCGGGCGCCGTGGATAGAG<br>CAGGAGGGGCCGGAGTATTGGGACCGGAACACACAGATCTACAAGGCCCAGGCACAGACTGACCGAGAGAG<br>CCTGCGGAACCTGCGCGGCTACTACAACCAGAGCGAGGCCGGTGAGTGACCCCGGCCCGGGGCGCAGGTCAC<br>GACTCCCCATCCCCCACGTACGGCCCGGGTCGCCCCGAGTCTCCGGGTCCGAGATCCGCCTCCCTGAGGCCGC<br>GGGACCCGCCCAGACCCTCGACCGGCGAGAGCCCCAGGCGCGTTTACCCGGTTTCATTTTCAGTTGAGGCCAA<br>AATCCCCGCGGGTTGGTCGGGGCGGGGCGGGGCTCGGGGGACTGGGCTGACCGCGGGGCCGGGGCCAGGGTC<br>TCACACCCTCCAGAGCATGTACGGCTGCGACGTGGGGCCGGACGGGCGCCTCCTCCGCGGGCATGACCAGTA<br>CGCCTACGACGGCAAGGATTACATCGCCCTGAACGAGGACCTGCGCTCCTGGACCGCCGCGGACACGGCGGC<br>T
HLA-B*5801	CGACACCCGTTCGTGAGGTTCGACAGCGACGCCGCGAGTCCGAGGACGGAGCCCCGGGCGCCATGGATAGAG<br>CAGGAGGGGCCGGAGTATTGGGACGGGGAGACACGGAACATGAAGGCCTCCGCGCAGACTTACCGAGAGAA<br>CCTGCGGATCGCGCTCCGCTACTACAACCAGAGCGAGGCCGGTGAGTGACCCCGGCCCGGGGCGCAGGTCAC<br>GACTCCCCATCCCCCACGTACGGCCCGGGTCGCCCCGAGTCTCCGGGTCCGAGATCCGCCTCCCTGAGGCCGC<br>GGGACCCGCCCAGACCCTCGACCGGCGAGAGCCCCAGGCGCGTTTACCCGGTTTCATTTTCAGTTGAGGCCAA<br>AATCCCCGCGGGTTGGTCGGGGCGGGGCGGGGCTCGGGGGACGGGGCTGACCGCGGGGCCGGGGCCAGGGT<br>CTCACATCATCCAGAGGATGTATGGCTGCGACCTGGGGCCCGACGGGCGCCTCCTCCGCGGGCATGACCAGTC<br>CGCCTACGACGGCAAGGATTACATCGCCCTGAACGAGGACCTGAGCTCCTGGACCGCGGCGGA<br>CACCGCGGCT

Table S2. Touchdown PCR protocol used for *HLA-B*5801* detection.

PCR step		Temperature (℃)	Time (seconds)
Initial denaturation		98	60
10 cycles	Denature	98	10
	Anneal	73 (− 1/cycle)	15
	Extend	68	30
25 cycles	Denature	98	10
	Anneal	63	15
	Extend	68	30

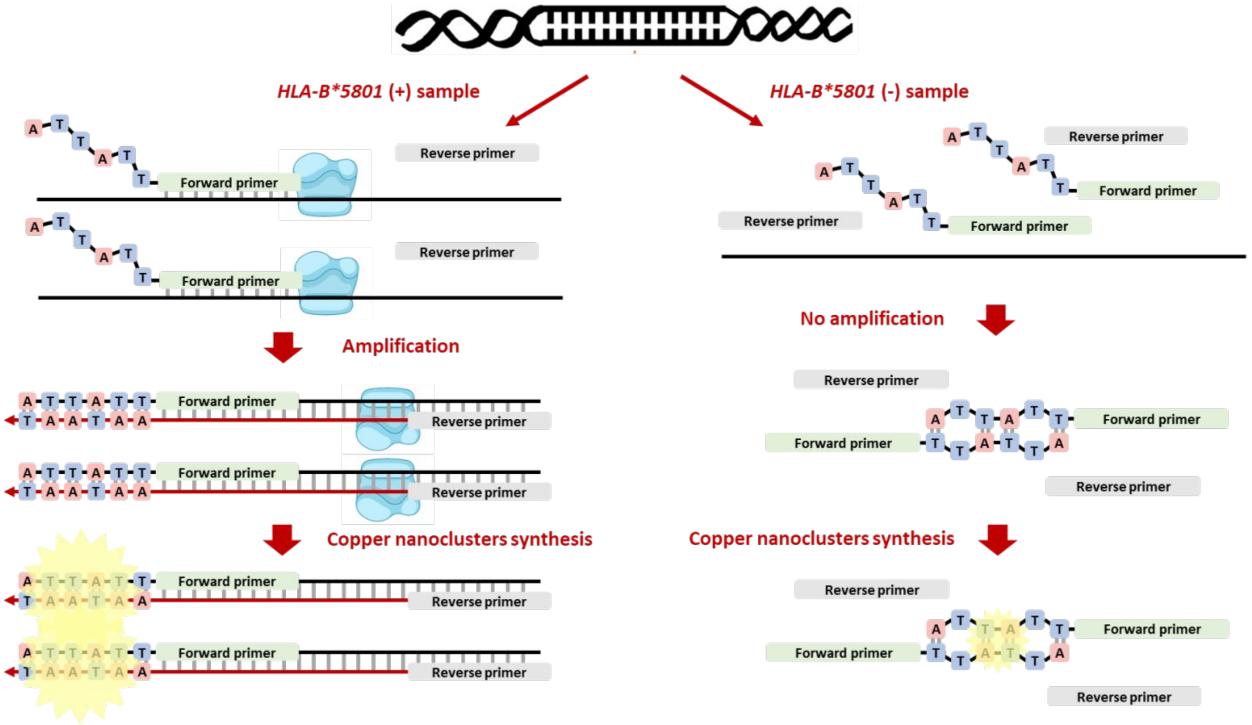

Figure S1. The mechanism for generating a single mismatch every three nucleotides utilizing a poly-ATT labeled primer.

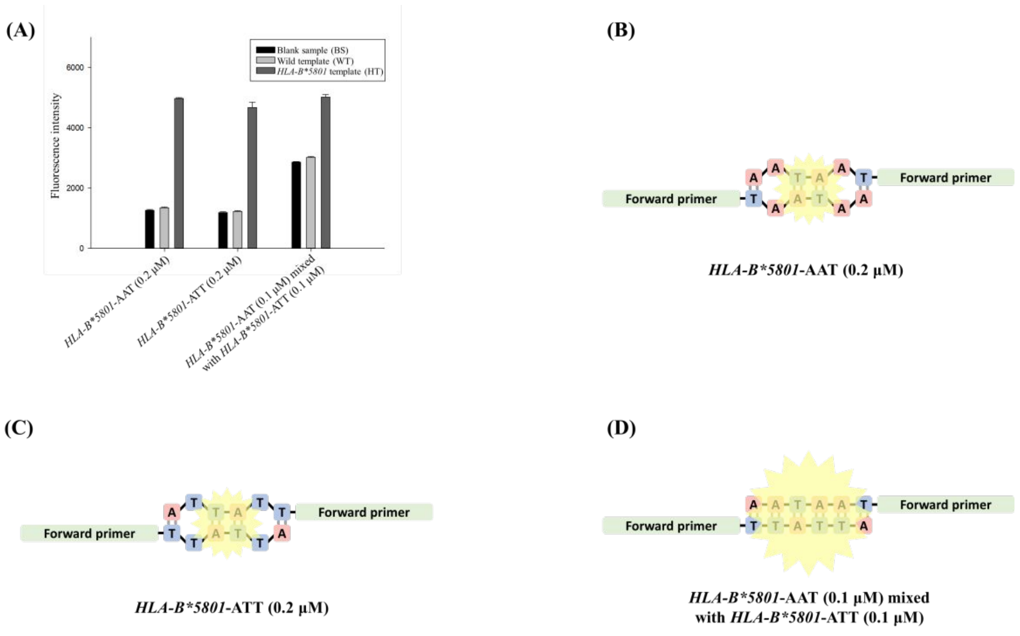

Figure S2. Supplementary evidence corroborating the mechanism of poly-AAT labeled primers. (A) Variations in fluorescence were recorded among the blank sample (BS), wild template (WT), and *HLA-B*5801* template (HT) using various primer combinations (n=3). (B) The self-annealing primer complex formed by poly-AAT primers alone. (C) The self-annealing primer complex formed by poly-ATT primers alone. (D) The self-annealing primer complex demonstrating no mismatches, achieved through the combination of poly-AAT and poly-ATT labeled primers.

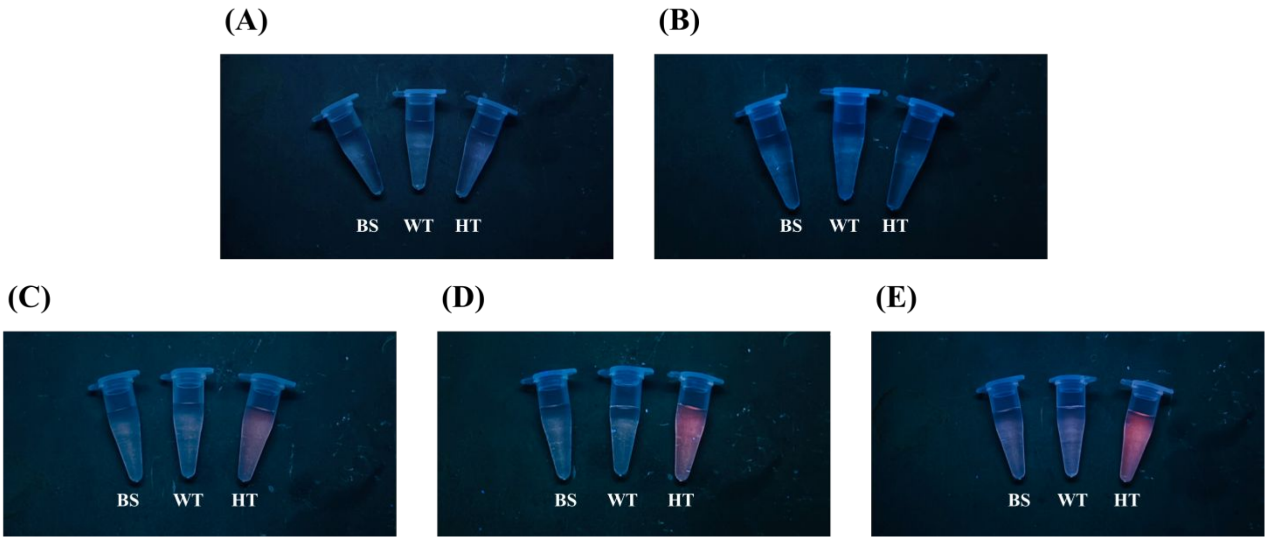

Figure S3. The fluorescence images of PCR products obtained under 365 nm UV light, as detected using the following forward primers: (A) *HLA-B*5801*-non (0-mer), (B) *HLA-B*5801*-AAT (15-mer), (C) *HLA-B*5801*-AAT (30-mer), (D) *HLA-B*5801*-AAT (45-mer), and (E) *HLA-B*5801*-AAT (60-mer). BS: Blank sample, WT: Wild template, HT: *HLA-B*5801* template.

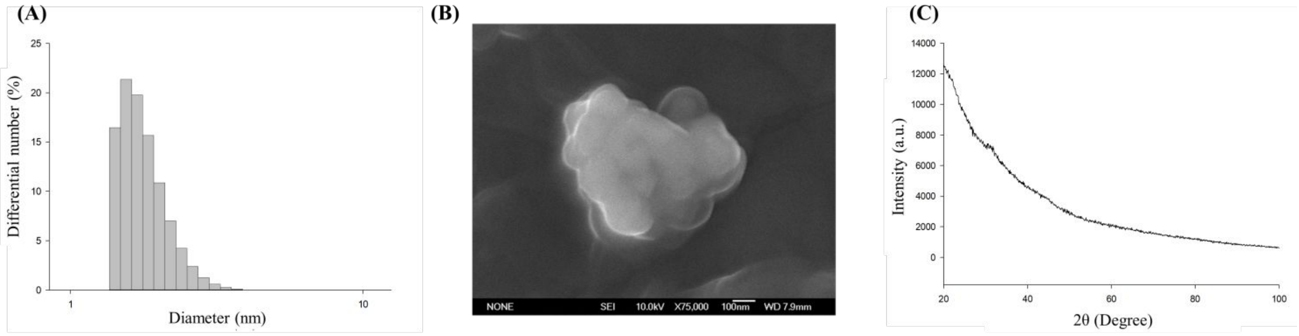

Figure S4. (A) Dynamic light scattering (DLS) results of the synthesized poly-AAT-templated copper nanoclusters. (B) Scanning electron microscopy (SEM) image of the synthesized poly-AAT-templated copper nanoclusters. (C) X-ray diffraction (XRD) patterns of the synthesized poly-AAT-templated copper nanoclusters.

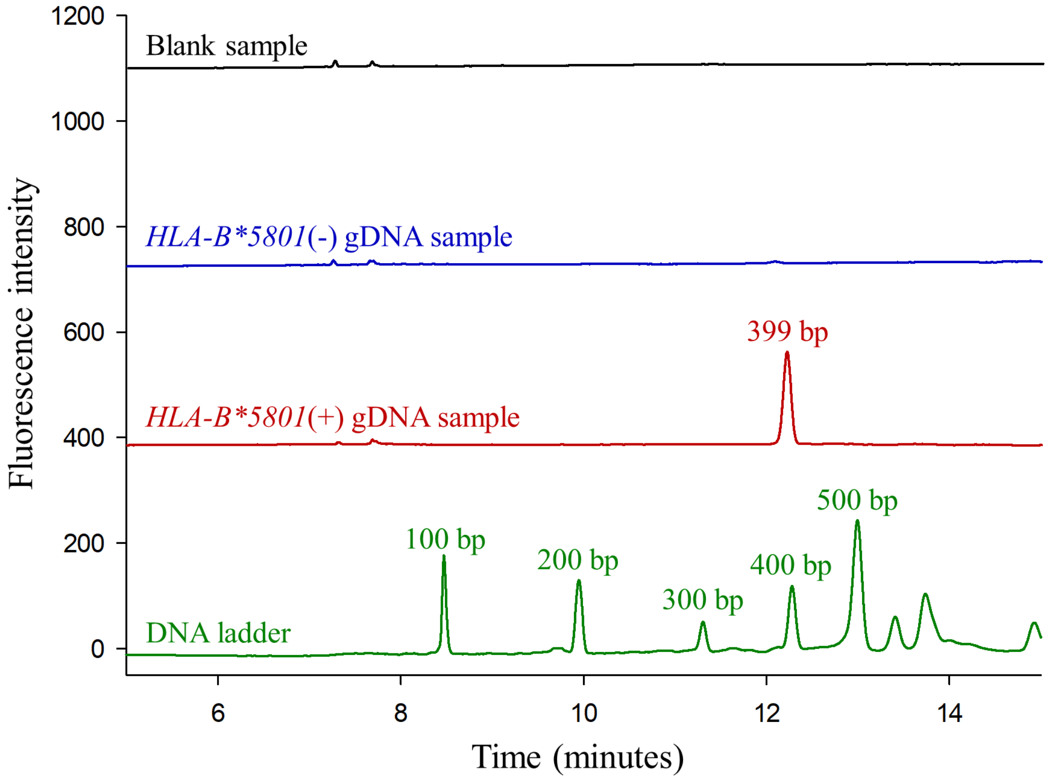

Figure S5. The capillary gel electrophoresis diagram of blank, *HLA-B*5801*(-) gDNA, *HLA-B*5801*(+) gDNA samples and DNA ladder.
